# Supplementary figures and images for: Effect of Winemaking on Phenolic Compounds and Antioxidant Activities of Msalais Wine
Source: Molecules. 2023 Jan 27;28(3):1250. doi: 10.3390/molecules28031250 (PMC9921378; doi:10.3390/molecules28031250)

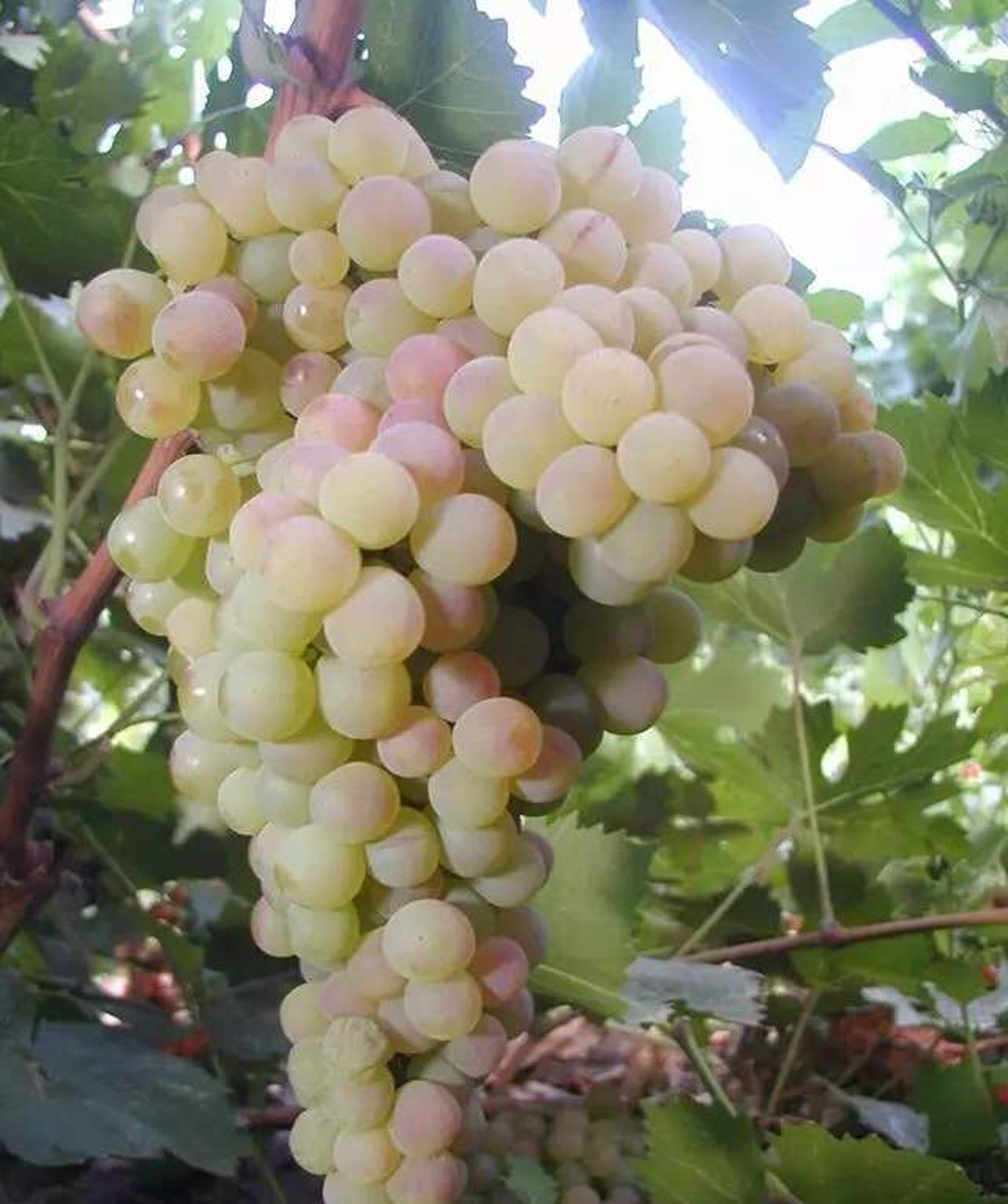

Supplement: Supplementary file 1 [file molecules-28-01250-s001.zip › Supplementary Figure S2.tif]
